# Dopamine Assisted One-Step Pyrolysis of Glucose for the Preparation of Porous Carbon with A High Surface Area

**DOI:** 10.3390/nano8100854

**Published:** 2018-10-19

**Authors:** Hanbo Xiao, Cheng-an Tao, Yujiao Li, Xianzhe Chen, Jian Huang, Jianfang Wang

**Affiliations:** College of Liberal Arts and Science, National University of Defense Technology, Changsha 410073, China; xiaohanbo16@nudt.edu.cn (H.X.); liyujiao@nudt.edu.cn (Y.L.); chenxianzhe13@nudt.edu.cn (X.C.); huangjian2015@nudt.edu.cn (J.H.)

**Keywords:** porous carbon, sugar-blowing, dopamine, glucose, one-step pyrolysis

## Abstract

Herein, a facile dopamine assisted one-pot synthesis approach is proposed for the preparation of porous carbon with a specific surface area (SSA) up to 2593 m^2^/g through the direct pyrolysis of a mixture of glucose, NH_4_Cl, and dopamine hydrochloride (DAH). The glucose is adopted as the carbon source and foaming agent, NH_4_Cl is used as the blowing agent, and DAH is served as collaborative carbon precursor as well as the nitrogen source for the first time. The effect of dopamine on the component, structure, and SSA of the as-prepared porous carbon materials are systematically studied. The moderate addition of dopamine, which influences the condensation and polymerization of glucose, matches better with ammonium salt decomposition. The SSA of porous carbon increases first and then decreases with the increasing amount of dopamine. In our case, the porous carbon produced with 5 wt% dopamine (PC-5) achieves the maximum SSA of up to 2593 m^2^/g. Accordingly, it also shows the greatest electrochemical performance. The PC-5 shows a capacitance of 96.7 F/g calculated from the discharge curve at 1 A/g. It also has a good capacitive rate capacity, the specific capacitance can still maintain 80%, even at a high current density of 10 A/g. Moreover, PC-5 exhibits a good cycling stability of 98.1% capacitive retention after 1000 cycles. The proposed method may show promising prospects for preparing porous carbon materials as advanced energy storage materials, storage, and catalyst supports.

## 1. Introduction

Three dimensional porous carbon (PC) materials with ultrahigh specific surface areas (SSA) have attracted much attention recently for their application in many fields, including absorption, separations, catalysis, and energy storage devices such as solar cells, fuel cells, Li-ion, Na-ion batteries, and supercapacitors [1,2,3,4,5,6,7,8,9,10,11,12,13,14,15,16]. Supercapacitors, also called electrochemical capacitors, are considered important (complementary to batteries) due to their high power density, fast charge-discharge rate, excellent cycling stability and more reliable safety [14,15]. Especially, electrical double layer capacitors (EDCLs), which are promising energy storage devices for an uninterruptible and high power supply, storing electric energy in an electric double layer formed by physisorption of electrolyte ions on the surface of each porous carbon electrode. The specific surface area (SSA) of the porous carbon materials is one of the key parameters to enhance the capacitance of EDCLs. In general, under certain other conditions, the higher the SSA of porous carbon materials, the larger the specific capacitance is. Therefore, the synthesis of porous carbon with high SSA is still an active area of research.

Till now, many novel methods have been developed to prepare unique porous carbon materials [2,17,18,19,20,21,22,23], but the most commonly used one is still the direct pyrolysis of carbon precursors, such as sugars [24,25,26,27,28,29]. Usually, the SSA of porous carbon produced by the direct pyrolysis of sugar in the presence of inorganic catalysts is less than 1000 m^2^/g. For example, Wang et al. described a sugar blowing method by utilizing the decomposition of ammonium salt to generate gas bubbles to form porous carbon with a strutted graphene structure and an SSA of about 1000 m^2^/g [24]. Then they extended the concept of ammonium-assisted chemical blowing to sucrose and household sugars to obtain porous carbon with SSAs of 710 m^2^/g [25]. Zhu et al. reported a facile sugar-blowing technique with NH_4_Cl as the gas template to generate 3D self-supported metal involved carbon nanostructures (CoO@Co/N-C), whose SSAs were only 551.1 m^2^/g [26]. Fechler reported a hydrothermal carbonization of glucose in salt-water mixtures resulting in highly porous carbonaceous materials with SSAs up to 650 m^2^/g [27]. Wei et al. demonstrated that the SSA of a sucrose derived carbon film with H_2_SO_4_ as the catalyst was 989 m^2^/g [28]. However, in most cases, in order to produce more highly porous carbon with an SSA as high as 1500–3000 m^2^/g, a further physical or chemical activation step is required to generate the desired additional micropores. Wei and coworkers utilized a simple physical activation process, annealing the produced carbon film at 900 °C in carbon dioxide to induce an interconnected open porosity within carbon [28]. After being activated for 2 h, the SSA of an activated carbon film increased to 1636 m^2^/g. Chang et al. prepared nitrogen-doped porous graphitized carbon with a high SSA of 2129.8 m^2^/g by urea-assisted chemical blowing and subsequent KOH activation [29]. Besides, using some special template/catalysis can also produce porous carbon with a high SSA. For instance, Strubel and coworkers have presented the synthesis of hierarchical porous carbons with distinctive microporosity inside the thin carbon walls leading to very high specific surface areas even exceeding 3000 m^2^/g based on self-made 20 nm ZnO nanoparticles [30]. However, these techniques required multiple steps to synthesize the template or catalyst in advance of the porous carbon synthesis.

Dopamine (DA) is one of the most important catecholamine neurotransmitters in the mammalian central nervous system. As we all know, it can self-polymerize at alkaline pH values and spontaneously deposit polydopamine conformal films on virtually any surface [31]. Due to its nontoxicity, extensive distribution, sustainability, excellent biocompatibility, and high carbonization yield, recently, dopamine has been proposed as an alternative carbon source in the synthesis and applications of carbon spheres, hollow carbon spheres, and yolk-structured carbon nanocomposites through polymerization and carbonization [32,33,34,35,36,37,38,39,40,41]. However, the use of monomeric dopamine as a co-precursor with sugar for the synthesis of porous carbon with a high surface area has not been explored.

**Figure 1:**
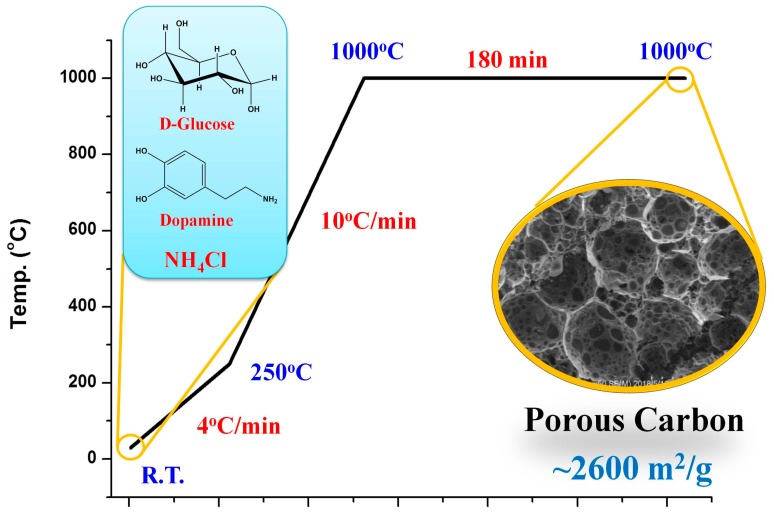
The illustration of the preparation of porous carbon.

In this study, we report a facile dopamine assisted one-pot synthesis technique for the preparation of porous carbon whose SSA can reach 2593 m^2^/g (Figure 1). This approach was realized by the direct pyrolysis of a mixture of glucose, NH_4_Cl, and dopamine hydrochloride (DAH), where glucose was used as the carbon source and foaming agent, NH_4_Cl was adopted as the blowing agent, and DAH was served as collaborative carbon precursor as well as the nitrogen source. The effect of dopamine on the component, structure, and SSA of the as-prepared porous carbon materials was systematically studied. The electrochemical performance of produced porous carbon materials as supercapacitor electrodes was also explored. To our knowledge, monomeric dopamine, which can be served as a co-precursor to produce highly porous carbon with SSAs up to about 2600 m^2^/g, is demonstrated for the first time.

## 2. Experimental Section

### 2.1. Materials

Glucose (AR) and dopamine hydrochloride (DAH, AR) were purchased from Sinopharm (Beijing, China), ammonium chloride (NH_4_Cl, AR) was received from Macklin reagent (Shanghai, China). All reagents were used as received without further purification.

### 2.2. Synthesis of Porous Carbon

Typically, 2 g of glucose was mixed with a specific amount of DAH (0 wt%, 1 wt%, 5 wt%, and 10 wt% to glucose, respectively) and NH_4_Cl. The weight ratio of NH_4_Cl to the mixture of glucose and dopamine was kept at 1:1. The mixture was ground for 30 min for blend, which was then heated to 250 °C at a rate of 4 °C/min, and then heated to 1000 °C at a rate of 10 °C/min and finally heated at 1000 °C for 3 h under a N_2_ atmosphere in a tube furnace (80 cm in length, 10 cm in diameter). Black foam-like products (porous carbon) were collected and denoted as PC-0, PC-1, PC-5, and PC-10, respectively.

### 2.3. Characterization

A thermogravimetric analysis (TGA) and differential thermal analysis (DTA) were done using a STA6000 thermogravimetric analyzer (PerkinElmer, Waltham, MA, USA) at a scanning rate of 4 °C/min under a N_2_ atmosphere from 30 °C to 700 °C. Scanning electron microscopy (SEM) images were obtained using an S-4800 electron microscope (Hitachi, East Coast Harbor, Honshu Island, Japan). The samples were sputtered with a thin layer of Au prior to imaging. Fourier transform infrared (FT-IR) spectra were collected using a Spectra Two spectrophotometer (PerkinElmer, Waltham, MA, USA) from 4000 cm^−1^ to 400 cm^−1^ with an attenuated total reflection (ATR) accessory. The N_2_ adsorption-desorption isotherm of the samples was measured at a liquid nitrogen temperature (78K) and gas saturation vapor tension range by a BEL Mini sorption instrument. The Brunauer–Emmett–Teller (BET) surface area was estimated over a relative pressure range of 0.05–0.3. The powder X-ray diffraction (XRD) patterns were carried out on a Ttr III type X-ray diffractometer (Rigaku, Tokyo, Japan) in θ-θ geometry from 5° to 50° (2θ) with a graphite-monochromated CuKα radiation source. Raman spectra were obtained on an RM2000 Raman spectrometer (Renishaw, Gloucestershire, UK) from 500 cm^−1^ to 3500 cm^−1^. Elemental analyses of C, H, and N were performed on a vario MACRO cube elemental analyzer (Elementar, Langenselbold, Germany). All electrochemical measurements were done in a 6 mol/L KOH aqueous electrolyte using a CHI660C (Shanghai Chenhua Apparatus, Shanghai, China) electrochemical workstation with a three-electrode experimental setup, where a platinum wire electrode and a saturated Ag/AgCl electrode were served as the counter and reference electrodes, respectively.

## 3. Results and Discussion

### 3.1. Synthesis of Porous Carbon

**Figure 2:**
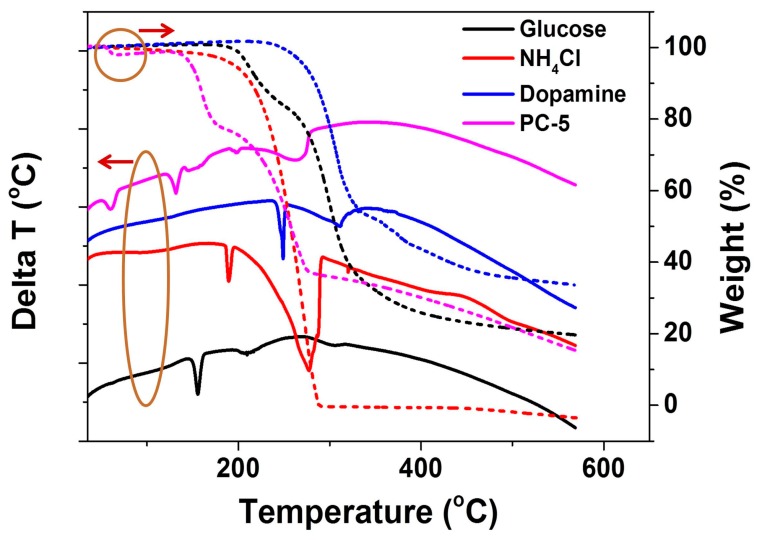
The DTA (differential thermal analysis) curves (solid line) and TGA (thermogravimetric analysis) curves (dash line) of glucose, NH_4_Cl, dopamine and their mixture (the precursor for PC-5).

Porous carbon could be synthesized through the direct pyrolysis of a glucose, DAH, and NH_4_ Cl mixture at a specific heating rate under an inert atmosphere, as shown in Figure 1. In this process, a melted viscous mixture of glucose and dopamine was gradually polymerized, whereas chemically released gases (NH_3_, HCl) from NH_4_Cl and DAH blew glucose-derived polymers, such as melanoidin, into numerous large bubbles, which featured sugar blowing. The bubble walls were gradually thinned by the gas release, the blowing, the surface-tension induced drainage of the polymer fluid out of the walls, and the elimination of small molecules from the polymers. The polymer walls were subsequently carbonized into an ultrathin sheet at a high temperature. The presence of dopamine reacted with glucose to form different glucose-derived polymers which affected the resulting carbon nanostructures. DTA and TGA were used to confirm the process. In the DTA profiles (Figure 2, solid lines), the three endothermic peaks during heating glucose alone corresponded to melting, water elimination, condensation, and polymerization. The two peaks of NH_4_Cl related to the crystal transformation and decomposition. Two endothermic peaks during heating dopamine hydrochloride alone corresponded to melting, condensation, and polymerization. In the pyrolysis of the mixture of glucose, dopamine hydrochloride, and NH_4_Cl, the reaction process is different. In the TGA curve of the PC-5 precursor (Figure 2, dash lines), the first mass loss occurs at 65 °C, which is much lower than that of the mixture of glucose and NH_4_Cl in the absence of DAH (130 °C) [24], presumably resulting from the water loss of the condensation of aldohexose and dopamine to N-substituted glucosylamine derivative. Then the mass loss occurs at 150 °C, presumably resulting from the water loss of the condensation of aldohexose and ammonia to N-substituted glucosylamine. Glucosylamine and its derivative become ketosamine derivatives after a quick cyclization and Amadori rearrangement (the 1st step of the Maillard reaction). In the DTA profile, the strong endothermic peak at 131 °C should result from melting, followed by a series of endothermic peaks (145–165 °C) which are dominated by the 2nd step of the Maillard reaction. In the 3rd step of the Maillard reaction, condensation and polymerization of the active intermediates produced in the 2nd step occurred. Diverse fragments such as nitrogenous heterocyclic compounds are lost. Meanwhile, the condensation and polymerization of dopamine and the decomposition of NH_4_Cl occurred, which contributed to the strongest mass loss noted around 250 °C. After that, the unstable O-containing and N-containing groups were lost slowly in the elevated temperature.

### 3.2. The Effect of Dopamine on Porous Carbon

**Figure 3:**
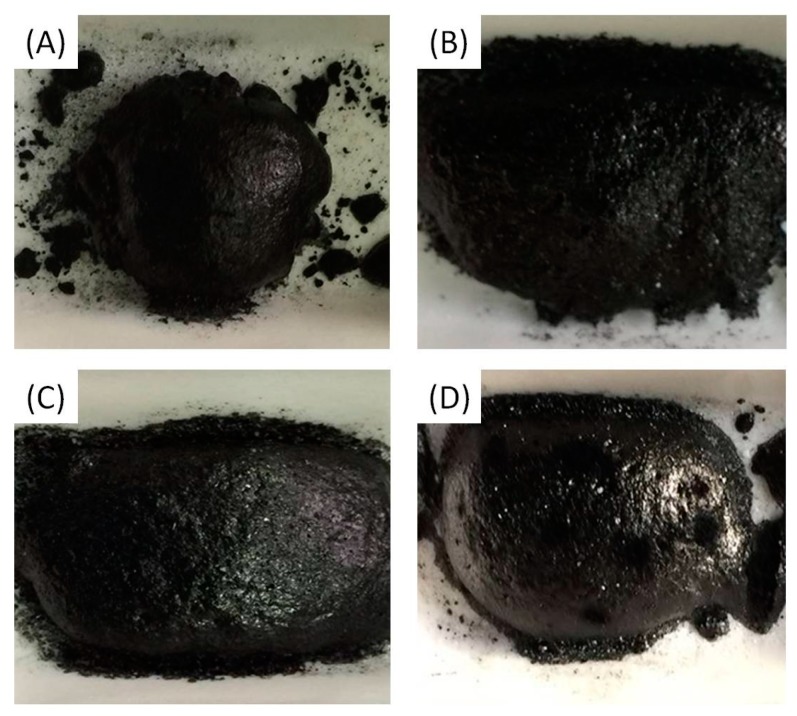
Photographs of porous carbon, (**A**) PC-0, (**B**) PC-1, (**C**) PC-5, (**D**) PC-10.

**Figure 4:**
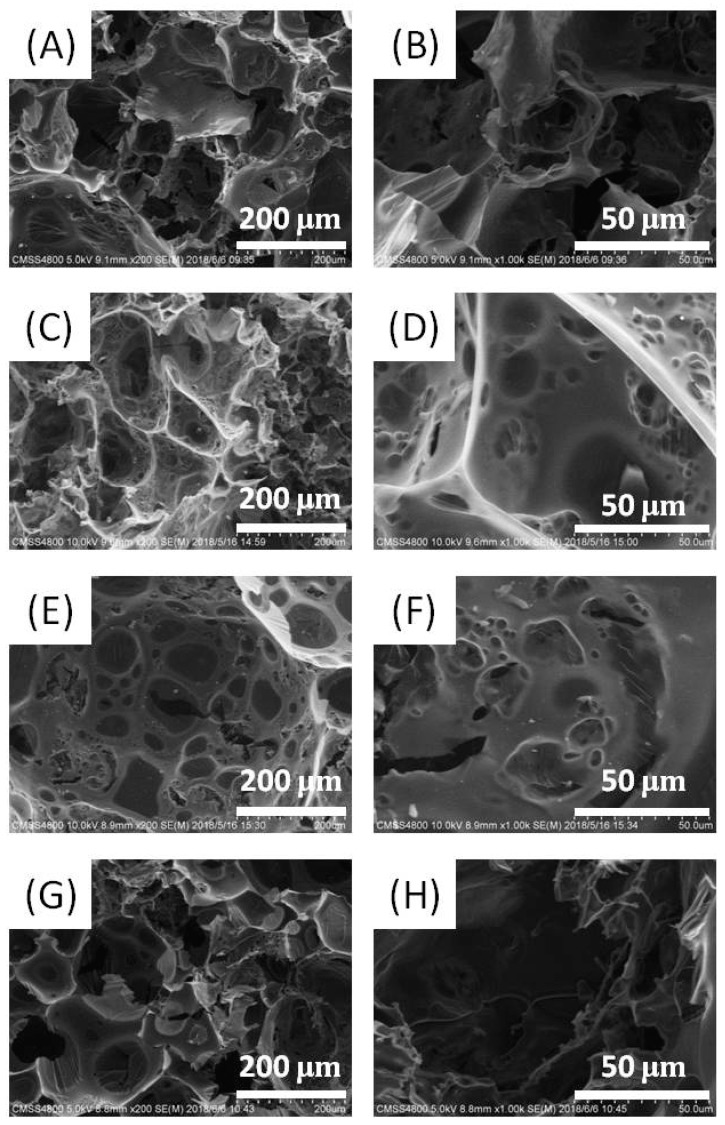
Typical SEM images of porous carbon, (**A**,**B**) PC-0, (**C**,**D**) PC-1, (**E**,**F**) PC-5, (**G**,**H**) PC-10.

To explore the effect of dopamine on porous carbon, the products obtained in the absence or presence of a varied amount of dopamine were characterized in detail. All products were black solids with a metallic luster (Figure 3). They showed certain compression-recovery elasticity (Figure S1). Typical SEM micrographs of the resulting samples were shown in Figure 4. They showed a foam-like architecture packed by large polyhedral bubbles. The amount of dopamine had an obvious effect on the morphology of the porous carbon. Without the dopamine assist, the cell size (bubble size) was very different, the cells seemed to be extruded, and their shapes were not regular. Under a 5 wt% dopamine-assist, the voids of PC-1 looked ellipsoidal, and their sizes were about 100 μm. There were many second-level pores in the wall of the cell. The PC-5 samples had the largest cells, their sizes were about 600 μm (Figure S2) and the shapes were very spherical. The second level pores in the wall were about 20–100 μm and there were still many third level pores (less than 5 μm) between the second level pores. By further increasing the amount of dopamine in the precursor, the cells in PC-10 cracked. Thus, the PC-5 had the optimized topological structure with the most abundant pores. The moderate addition of dopamine, which influenced the condensation and polymerization of glucose, better matched the ammonium salt decomposition.

**Figure 5:**
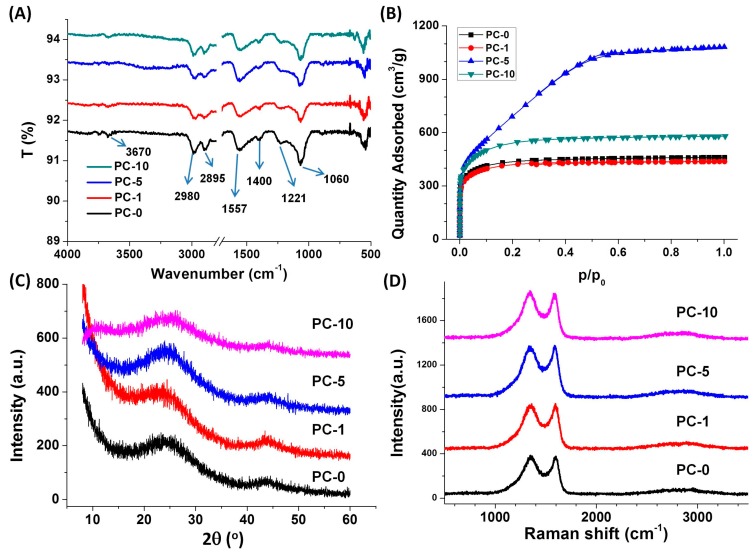
(**A**) The FT-IR (Fourier transform infrared) spectra, (**B**) N_2_ adsorption-desorption isotherms, (**C**) XRD (X-ray diffraction) patterns, (**D**) The Raman spectra of the porous carbon materials.

The FTIR characterization gave some information on the chemical structure of porous carbon (Figure 5A). Compared to the IR spectra of glucose and dopamine (Figure S3 and S4), the broad band around 3670 cm^−1^, which was assigned to the N−H and O−H stretching mode, nearly disappeared, indicating that most of the O-containing and N-containing groups had been removed after carbonization. The peaks at 2980 cm^−1^ and 2895 cm^−1^ were related to the C−H stretching mode, and the peak at 1060 cm^−1^ was attributed to the C−H in-plane deformation, suggesting the existence of hydrogen residue after carbonization. The peak at 1557 cm^−1^ was assigned to the stretching of aromatic C−C bonds of carbon. The peak at 1400 cm^−1^ was attributed to the heterocyclic stretching, while the peak at 1221 cm^−1^ was attributed to the phenol C–OH stretching, indicative of the existence of O-containing group residue. Due to the low addition of dopamine, the products, even PC-10, did not show any special absorption peaks of dopamine derivatives (for example, 1507 cm^−1^ of the C−N bending, 1157 cm^−1^ of the heterocyclic N−H in-plane deformation breathing).

To further study the effect of dopamine on the SSA of carbon foam, we conducted N_2_ adsorption-desorption isotherm measurements on four carbon foam samples with different amounts of dopamine. Calculated from the curves shown in Figure 5B, the BET SSAs of PC-0, PC-1, PC-5, and PC-10 products were around 1599 m^2^/g, 1605 m^2^/g, 2593 m^2^/g, and 2026 m^2^/g, respectively. It indicated that the addition of dopamine in the precursor led to a higher SSA of the resulting porous carbon. The SSA of porous carbon increased first and then decreased with the increasing amount of dopamine. In our case, the maximum SSA that PC-5 could achieve up to about 2600 m^2^/g, which was near to the multistep activated carbon. The total pore volume of PC-5 reached 1.67 cm^3^/g, and the average pore size was the biggest one (Table 1). As shown in Figure 5B, PC-0, PC-1, and PC-10 had a type I isotherm with no hysteresis, while the PC-5 carbon foam started to show a little hysteresis but still kept the type I shape, indicating its microporous structure. The pore size distributions calculated using the Barrett–Joyner–Halenda (BJH) method were shown in Figure S5. The pore size of PC-5 was about 1 nm, while the other samples had no pores whose size was more than 0.8 nm. As we know, BJH could be applied to mesoporous distribution analysis, but was not suitable for a micropore filling description. Then the pore size distributions were analyzed using the MP method again, as shown in Figure S6. Specifically, the pore size distributions in PC-5 were mainly about 0.6 nm and 1.2 nm, while the other samples had no pores with a size larger than 1 nm. The result was in agreement with the SEM observation.

To confirm the graphitized structure of as-prepared porous carbon materials, XRD and Raman patterns were performed. Figure 5C showed the XRD patterns of the as-prepared porous carbon samples. Two broad diffraction peaks at 2θ = 24° and 44°, which corresponds to (002) and (100) planes of graphite carbon could be observed, which indicates the partial formation of graphitized carbon framework. The intensity of diffraction peak in PC-5 was only a little higher than those of other samples, suggesting the addition of dopamine had no evident effect on the degree of graphitization. Raman spectroscopy was a noninvasive technique for the characterization of structural and electronic properties of carbon-based materials [42]. Figure 5D presented the Raman spectra of porous carbon. For all samples, there were two dominating peaks at 1350 cm^−1^ and 1584 cm^−1^, corresponding to D band and G band, respectively. The D band was related to A_1g_ breathing mode of *sp*^3^ carbon, and the G peak corresponded to the in-plane bond-stretching of all pairs of C *sp*^2^ atoms in both rings and chains [43,44,45]. Such feature of Raman spectrum typically meant the appearance of the mixture of amorphous carbon and nanocrystalline graphite. The 2D band at ~2700 cm^−1^ relating to the stacking order of the graphitic structure along the c axis was very low, due to the relative low carbonization temperature (1200 °C) [42]. The elemental compositions of as-prepared porous carbon materials were analyzed by elemental analysis, as shown in Table 1. The content of N increases by increasing the amount of dopamine, mainly due to and the N from dopamine remained on the carbon materials after pyrolysis. There were still over 20% O remained after pyrolysis, indicating the presence of O-containing groups (commonly heterocyclic O and OH groups) in the porous carbon materials, which was in accordance with the IR results.

### 3.3. Electrochemical Performance of Porous Carbon

**Figure 6:**
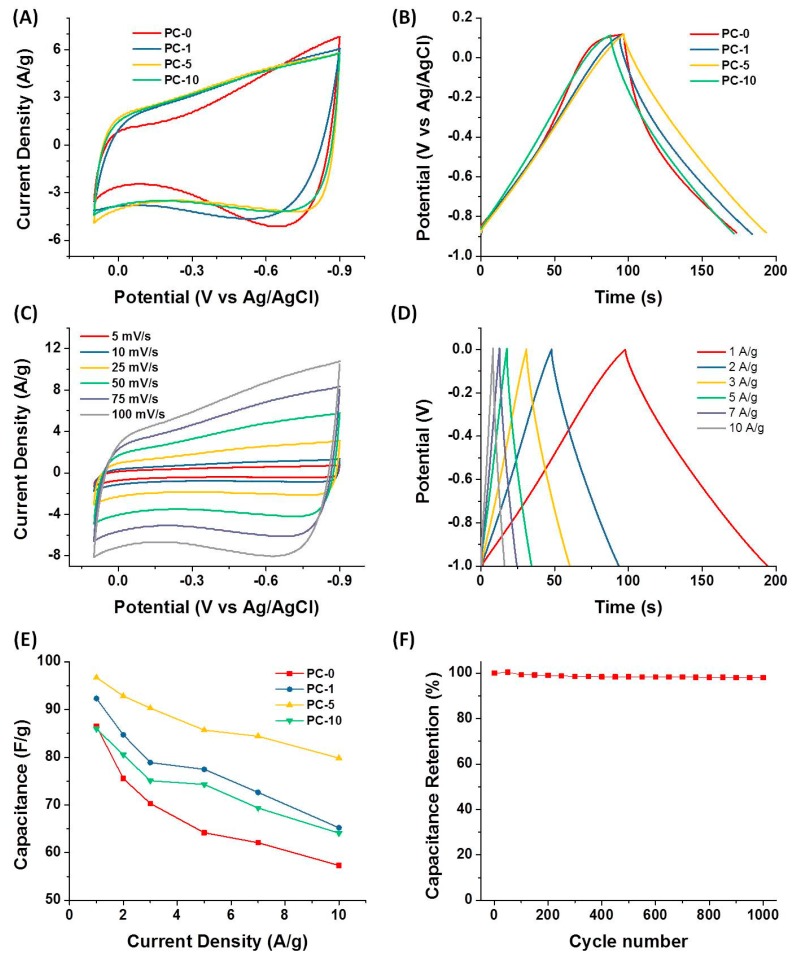
(**A**) The cyclic voltammogram (CV) curves of all porous carbon materials at the scan rate of 50 mV/s measured in a three-electrode system; (**B**) Galvanostatic charge/discharge (GCD) curves of all porous carbon materials at 1 A/g measured in a three-electrode system; (**C**) CV curves of PC-5 electrode material at different scan rates measured in a three-electrode system; (**D**) GCD curves of the PC-5 electrode at different current densities measured in a three-electrode system; (**E**) specific capacitances of all porous carbon electrode materials at different current densities in a three-electrode system; (**F**) Cycling stability measured for PC-5 at 1 A/g measured in a three-electrode system.

Electrochemical performances of as-synthesized porous carbon materials as supercapacitor electrodes were evaluated by a three-electrode system in a 6 M KOH aqueous electrolyte. The cyclic voltammogram (CV) curves of all the porous carbon samples at a scan rate of 50 mV/s were shown in Figure 6A. They exhibited a quasi-rectangular shape in a potential range of −0.9 V to 0.1 V, manifesting the approximately ideal electric double-layer capacitive (EDLC) behavior. There was a slight hump around −0.7 V indicative of the pseudo-capacitance dedicated by the doped nitrogen atoms [46]. It has been reported that nitrogen doping could not only enhance the EDLC by changing the electron donor/acceptor of carbon materials and improve the wettability between electrolyte and electrode materials [47], but also bring pseudo-capacitance effects by participating in a pseudo-faradaic charge-transfer reaction [13]. Apparently, the CV curve of PC-5 encircled the largest curve area among all the electrodes, suggesting the highest capacitance value, mainly due to their largest SSA.

Figure 6B compares the galvanostatic charge/discharge (GCD) curves of all PC electrode materials at 1 A/g current density, which was a more accurate method to measure the capacitance value. All GCD curves show a nearly linear and symmetrical with a slight curvature shape, implying good electrical double-layer capacitive behavior with little pseudo-capacitance. Similarly, the PC-5 electrode showed the longest discharge time, meaning it had a larger capacitance value than other samples, which was in agreement with the result above the CV curves. Furthermore, the CV and GCD curves of PC-5 over various scan rates and current densities were systematically investigated. All CV curves (Figure 6C) retained a quasi-rectangular shape, suggesting the approximately ideal EDLC nature. More importantly, the CV curves kept the quasi-rectangular shape even at a high scan rate of 100 mV/s, suggesting the efficient ions transfer and the fast charge response capacity inside the PC-5 electrode. All GCD curves of PC-5 (Figure 6D) exhibited a quasi-triangular and symmetrical appearance, indicating excellent super-capacitive behavior and electrochemical reversibility, which was consistent with the result of the CV curves. The specific capacitance of the PC-5 electrode was 96.7 F/g calculated from the discharge curve at 1 A/g. Besides, the voltage drop at the initiation of the discharge at a high current density of 10 A/g was only about 0.01 V, implying a low internal resistance, which should benefit from the graphitized carbon structure, endowing the prominent electric conductivity. To determine the capacitive rate capacity, the correlations between specific capacitance and current densities of all PC electrodes were shown in Figure 6E. The capacitance value gradually decreased with the increase of the current density, which was related to the increase of the diffusion limitation. The PC-5 not only had the largest specific capacitance, but also had the best rate capacity among all porous carbon samples. The specific capacitance could still maintain itself at 80%, even at a high current density of 10 A/g, benefiting from their great porosity [29]. Cycling performance was another important factor in determining the supercapacitor electrodes for many practical applications. The cycling stability of PC-5 was examined using GCD cycling at a current density of 1 A/g (Figure 6F). During the 1000 cycles, the specific capacitances were almost constant (98.1% capacitive retention after 1000 cycles), which demonstrated good cycling performance.

**Figure 7:**
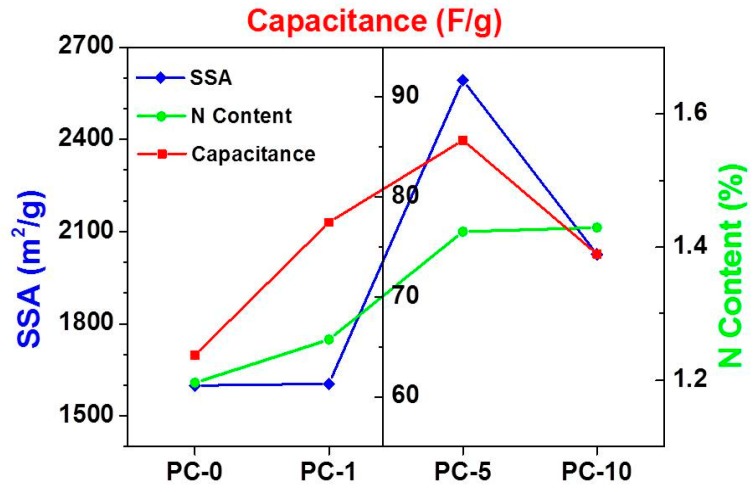
The correlations of SSA (specific surface area), the N content, and the specific capacitance at 5 A/g of the samples.

It is well known that there are two important factors that influence the specific capacitance of porous carbon materials. The first is the amount of doped heteroatoms, such as nitrogen, which is capable of providing pseudo-capacitance. The second is the SSA of the materials, which is the key factor for the accommodation of electrolyte ions [43]. By increasing the content of dopamine in the precursor mixture, the varying trend of SSA, N content, and capacitance become visible, as shown in Figure 7. The SSA of porous carbon increases first and then decreases with the increasing amount of dopamine. The trend of capacitance changes exactly the same as the trend of SSA. Although the content of N increases by increasing the amount of dopamine, the content of N is generally small in all samples, and the corresponding pseudo-capacitance is also small. The specific capacitance of the porous carbon material is determined by the specific surface area of the porous carbon materials.

## 4. Conclusions

We have demonstrated a facile dopamine assisted one-pot synthesis method for the preparation of porous carbon with a high SSA for the first time. This approach used the direct pyrolysis of a mixture of glucose, NH_4_Cl, and dopamine hydrochloride, where glucose was used as the carbon source and foaming agent, NH_4_Cl was adopted as the blowing agent, and dopamine hydrogen chloride served as the collaborative carbon precursor as well as the nitrogen source. The dopamine had an evident effect on the component, structure, SSA, and electrochemical behaviors of the as-prepared porous carbon materials. The moderate addition of dopamine, which influences the condensation and polymerization of glucose, could induce a better match with ammonium salt decomposition. The SSA of porous carbon increased first and then decreased with the increasing amount of dopamine. In our case, the porous carbon produced with 5 wt% dopamine could achieve a maximum SSA of up to about 2600 m^2^/g. Accordingly, they showed high capacitance, good rate capacity, and cycling stability mainly due to their large SSA. The proposed method may show promising prospects for preparing porous carbon materials as advanced energy storage materials, storage, and catalyst supports.

## Figures and Tables

**Table 1:** The specific surface area, pore structures, elemental analysis, and carbon yield of porous carbon.

Samples	S_BET_ (m^2^/g)	V_total_ (cm^3^/g)	Average Pore Size (nm)	N (%)	C (%)	H (%)	Yield (%)
PC-0	1599	0.71	1.78	1.196	73.03	2.679	49.7
PC-1	1605	0.68	1.69	1.261	73.27	2.563	51.4
PC-5	2593	1.67	2.58	1.423	75.73	2.507	65.7
PC-10	2026	0.90	1.77	1.429	75.48	2.309	58.2

## Supplementary Materials

The following are available online at http://www.mdpi.com/2079-4991/8/10/854/s1, Figure S1: Compression-recovery elasticity of PC-5. (A) Initial state of PC-5, (B) Pressed with a 200g weight, (C) In recovery and (D) After the recovery, Figure S2: FT-IR spectra of glucose, Figure S3: FT-IR spectrum of dopamine, Figure S4: Scanning electron microscopy image of PC-5 (50×), Figure S5: Barrett-Joyner-Halenda (BJH) analysis of porous carbon, (A) PC-0, (B) PC-1, (C) PC-5, (D) PC-10, Figure S6: MP analysis of porous carbon, (A) PC-0, (B) PC-1, (C) PC-5, (D) PC-10.
