# Supplementary material for: Dopamine Assisted One-Step Pyrolysis of Glucose for the Preparation of Porous Carbon with A High Surface Area
Source: Nanomaterials (Basel). 2018 Oct 19;8(10):854. doi: 10.3390/nano8100854 (PMC6215113; doi:10.3390/nano8100854)
Supplement: Supplementary file 1 [file nanomaterials-08-00854-s001.pdf]

Supporting Information

Dopamine Assisted One-Step Pyrolysis of Glucose for the Preparation of Porous Carbon with A High Surface Area

Hanbo Xiao †, Cheng-an Tao *,†, Yujiao Li, Xianzhe Chen, Jian Huang and Jianfang Wang *

College of Liberal Arts and Science, National University of Defense Technology, Changsha 410073, China; xiaohanbo16@nudt.edu.cn (H.X.); liyujiao@nudt.edu.cn (Y.L.); chenxianzhe13@nudt.edu.cn (X.C.); huangjian2015@nudt.edu.cn (J.H.)

† These authors contribute to this work equally.

* Correspondences: tca02@mails.thu.edu.cn (C.T.); wangjianfang@nudt.edu.cn (J.W.); Tel.: +86-731-8457-4241 (J.W.)

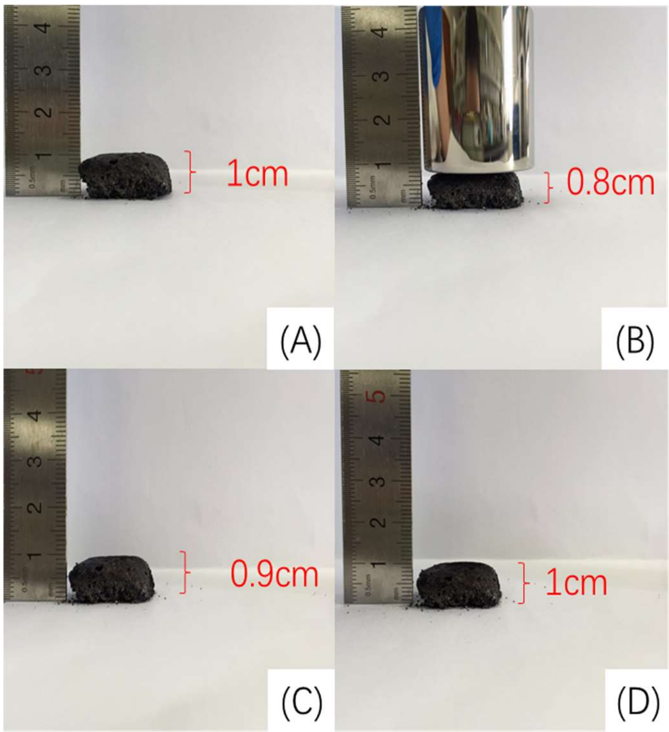

Figure S1. Compression-recovery elasticity of PC-5. (**A**) Initial state of PC-5, (**B**) Pressed with a 200g weight, (**C**) In recovery, and (**D**) After the recovery.

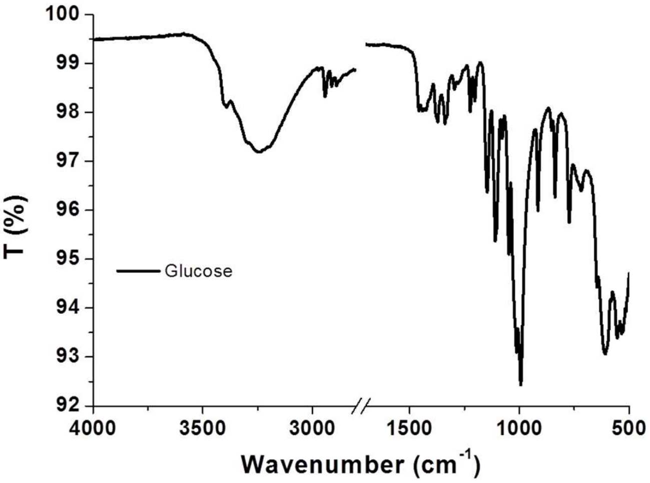

Figure S2. FT-IR (Fourier transform infrared) spectrum of glucose.

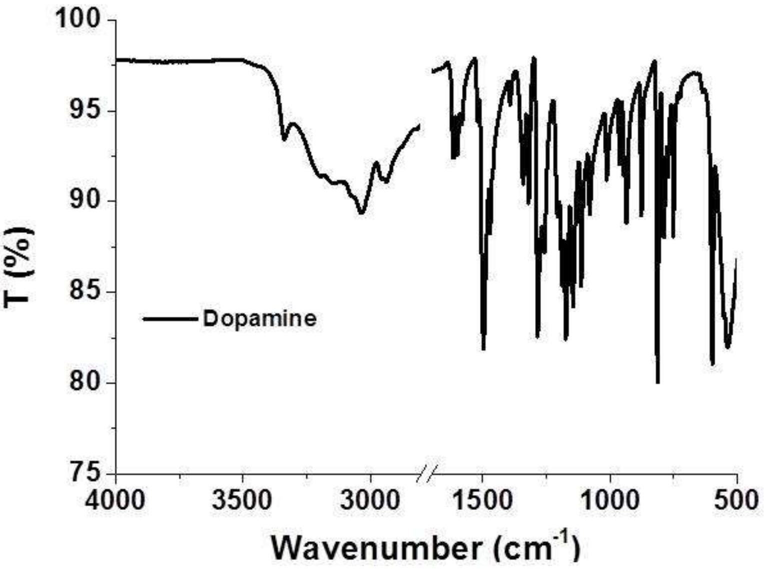

Figure S3. FT-IR (Fourier transform infrared) spectrum of dopamine.

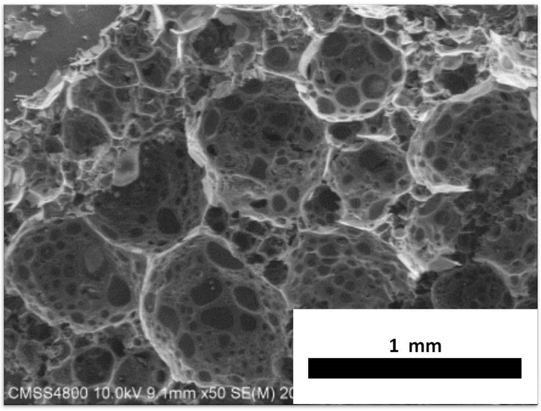

Figure S4. Scanning electron microscopy image of PC-5 (50×).

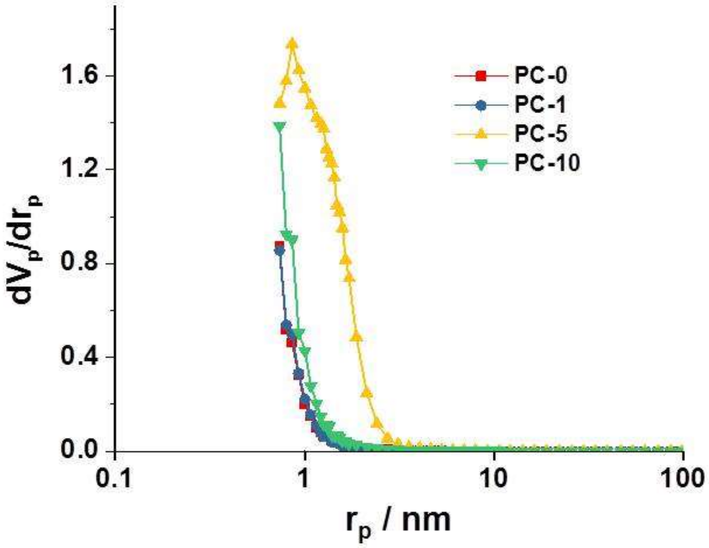

Figure S5. Barrett-Joyner-Halenda (BJH) analysis of porous carbon, (**A**) PC-0, (**B**) PC-1, (**C**) PC-5, (**D**) PC-10.

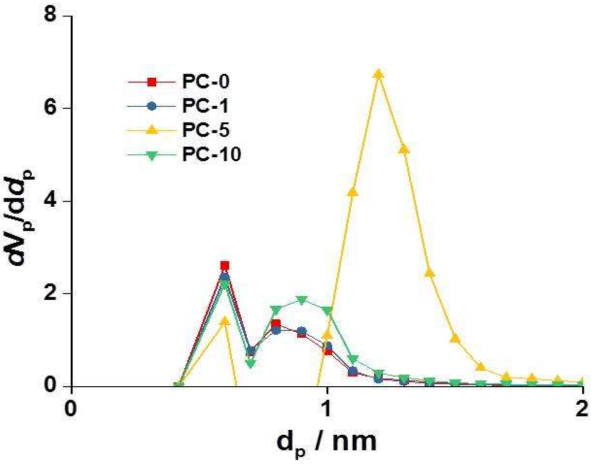

Figure S6. MP analysis of porous carbon, (**A**) PC-0, (**B**) PC-1, (**C**) PC-5, (**D**) PC-10.
